# Comparing Durations of Different Countermeasure Efficacies Against Wild Boar (*Sus scrofa*) in Cornfields of Hunchun, Jilin Province, China

**DOI:** 10.3390/ani15071017

**Published:** 2025-04-01

**Authors:** Ke Li, Bruce R. Burns, Shuang Cui, Qi Song, Chengxi Zhao, Mingtian Zhang, Dan Zhang, Bingwan Liu

**Affiliations:** 1 College of Wildlife and Protected Area, Northeast Forestry University, Harbin 150040, China; li_ke1201@163.com (K.L.);; 2 School of Biological Sciences, University of Auckland, Auckland 1142, New Zealand; b.burns@auckland.ac.nz

**Keywords:** human–wildlife conflict, Amur tiger, solar blinker, electric fencing, prevention and control effect, validity period, cost-effectiveness

## Abstract

Wildlife behavior can be influenced by the deployment of sensory cues in a landscape, but different cues vary in the strength and duration of their effectiveness. We aimed to identify the most effective and cost-efficient countermeasures (sensory cues) to deter wild boar (*Sus scrofa*) entry and damage to cornfields in Hunchun, Jilin Province, China. These cornfields have experienced severe damage by wild boars during the critical 30-day period when this crop was ripening. From 2016 to 2021, different countermeasures were applied sequentially seeking to control this damage by using either (1) visual deterrents, i.e., solar blinkers of different colors; (2) auditory deterrents, i.e., playbacks of Amur tiger (*Panthera tigris altaica*) calls, wild boar calls, or wolf (*Canis lupus*) calls; (3) tactile deterrents, i.e., electric fencing; (4) olfactory deterrents, i.e., Adult Amur tiger feces; or (5) various combined deterrents. We first evaluated the effectiveness of these broad categories, then performed a detailed analysis of the individual countermeasures to assess their specific deterrence effectiveness and duration. A cost-effective analysis was subsequently performed on the most effective countermeasures to evaluate the best option for practical applications. Across the broad categories of deterrents, the tactile group proved the most effective overall. For individual deterrents, the seven countermeasures showing significantly higher effectiveness than the others tested included the following: (1) 1000 mA red solar blinker (32.25 ± 4.22 days), (2) 1000 mA yellow solar blinker (29.67 ± 4.58 days), (3) 1000 mA green solar blinker (29.58 ± 5.60 days), (4) electric fencing with three wires (29.67 ± 0.58 days), (5) electric fencing with two wires (28.00 ± 2.00 days), (6) Adult Amur tiger calls for 15 s and wild boar calls for 15 s plus a combined 30 s plus a blank recording for 5 min (26.50 ± 2.38 days), and (7) Adult Amur tiger feces and calls (27.34 ± 2.94 days). Except for the Adult Amur tiger feces and calls, each countermeasure would cover most of the period over which control is necessary (30 days). The 1000 mA red solar blinker of achieved the highest repellency per cost ratio (0.31) at 30.29 IUS$/hm^2^ but showed reduced effectiveness over time. Although electric fencing with three wires offers longer deterrence, its cost-effectiveness ratio was lower (0.27) due to higher installation and maintenance costs at 319.69 IUS$/hm^2^. The 1000 mA red solar blinker offers a highly cost-effective short-term deterrent, while the electric fencing with three wires provides durable, long-term protection despite its higher costs. Balancing cost and duration can optimize wild boar deterrence strategies across different management needs.

## Simple Summary

Wild boars cause substantial damage to cornfields in Hunchun, Jilin Province, particularly during the critical 30-day ripening period. To identify effective and cost-efficient deterrents, this study evaluated visual (solar blinkers), auditory (predator calls), tactile (electric fences), olfactory (tiger feces), and combined deterrent methods from 2016 to 2021. The results indicated that electric fences provided the most reliable long-term protection, preventing crop damage for up to 29 days. Solar blinkers, particularly red ones, were a cost-effective short-term solution but declined in effectiveness as the wild boars habituated to them. Predator calls, such as those of Adult Amur tigers, also demonstrated efficacy, likely due to the natural threat perception of wild boars. While electric fences require a higher initial investment, they offer sustained protection, making them suitable for long-term use. In contrast, solar blinkers provide a practical, low-cost alternative for short-term mitigation. These findings offer valuable insights for developing effective wildlife management strategies, helping to reduce agricultural losses while promoting human–wildlife coexistence.

## 1. Introduction

Conflicts between humans and wildlife have been reported worldwide [1,2]. Due to increasing human demands for land, wildlife habitats have diminished, and food resources have been strained, leading to a rise in human–wildlife conflicts [3,4]. Concurrently, initiatives, such as natural forest conservation, farmland reforestation [5,6], and the establishment of nature reserves, have improved wildlife habitats and facilitated population recovery, exacerbating these conflicts. Problems include attacks by predators on livestock, transmission of diseases from wild populations to domestic animals and humans, agricultural damage, and collisions with vehicles [7,8,9]. While no prevention and control measures have fully resolved human–wildlife conflicts, efforts have focused on mitigating their impacts [10].

Crop damage caused by wild boars (*Sus scrofa*) is one of the most widespread and persistent human–wildlife conflicts globally, with the potential to increase further [11,12,13,14]. Managing wild boar populations is a priority both within China and internationally. It is noteworthy that commonly employed management strategies have predominantly relied on lethal measures, such as hunting and poisoning [15]. In Texas, USA, a low dose of 0.01% and 0.005% warfarin in paraffin baits has successfully reduced wild boar populations [16]. In Oklahoma, USA, trapping has decreased wild boar damage to pastures by 90% [17]. Furthermore, approaches in Italy’s Castello della Borziano Nature Reserve and the Forest of Dean in the UK have achieved control goals through hunting and fertility management, significantly halving the time needed to reach wild boar population reductions compared to hunting alone [18]. Various measures have been implemented in China to control wild boar damage, such as fencing, digging trenches, buffer zones, disturbances, and human patrols. While chemical repellents and fumigants have shown promise, they remain cost-prohibitive for widespread use [19]. Thus, the choice of countermeasure depends not only on the desired reduction in wild boar damage, but also on the expenditures required for implementing these countermeasures [20].

Rather than simply controlling population size, an alternative strategy to managing wildlife is to deploy deterrents so that the wildlife will not enter sensitive areas [21]. This strategy depends on the animals detecting and responding to sensory cues in their environment [22]. Sensory cues include visual, auditory, tactile, and olfactory stimuli that animals use to make decisions and guide their movements. For wild boars, a range of sensory cues have been tested as repellents [23,24]. For instance, auditory deterrents, such as predator vocalizations (e.g., wolves howling or dogs barking), have reduced the wild boar’s presence and foraging activity in agricultural fields, mitigating crop damage in Södermanland County, Sweden [25]. Olfactory repellents, such as predator-derived scents, have effectively reduced wild boar damage to crops, with a sustained deterrent effect observed over 30 days in South Korea [26]. Furthermore, gustatory repellents, such as the phosphorous acid-based “SUCROSAN^®®^” pellets, showed a slight reduction trend in wild boar damage in Canton Basel-Land, Switzerland. However, they did not achieve significant damage prevention or area avoidance levels, indicating the potential for further exploration in crop protection strategies [27]. Therefore, the most cost-effective repellent remains unclear.

In Hunchun District, Jilin Province, wild boar damage to crops has been a significant issue. According to Hunchun Forestry Bureau statistics, from 2011 to 2017, wild boar damage to cornfields in Hunchun District resulted in losses exceeding CNY 8.62 million, with over 1519 hectares affected [28]. In 2011 alone, 428 incidents of wild boar crop damage were recorded, impacting 216.03 hectares of cornfields [29]. The most severe damage occurred in 2016, when 294.33 hectares of farmland were affected [29]. In 2017, wild boar damage accounted for 51.1% of the area of the total damaged cornfields [30].

Residents have employed various preventative measures, including trenches, disturbances, fencing, and nocturnal patrols, with the latter proving the most effective. Researchers have further experimented with deterrents, such as Adult Amur tiger feces, calls and images, wild boar calls, and wolf calls, as well as solar blinkers and electric fencing, achieving notable success [28,29,31,32,33]. The relative effectiveness of these different strategies, however, has not been compared. Our study aims to assess these alternatives based on their duration of effect and cost-effectiveness.

## 2. Materials and Methods

### 2.1. Study Site

**Figure 1:**
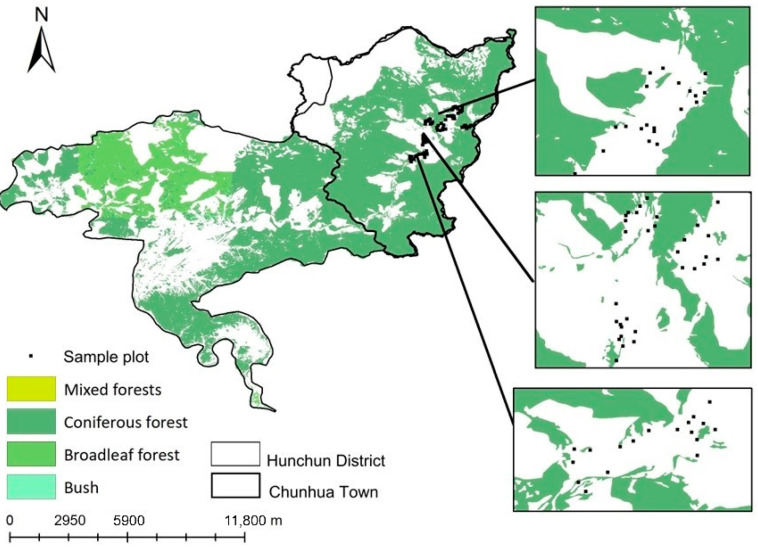
Map of sampling plot arrangement. Chunhua town is part of Hunchun district, and experimental plots were selected in cornfields located near forest edges, where wild boar damage was most severe. Due to the large geographic scale, sampling plots overlapped and were indistinguishable. Enlarged sections highlight the spatial arrangement of countermeasures and their distances from each other and the forest edge.

The study site was in Chunhua town, Hunchun District, Jilin Province, China (43°11′44″ N, 131°04′24″ E) (Figure 1), within the northeastern mountainous region of Yanbian Korean Autonomous Prefecture. Hunchun District, with an area of 5141 km^2^, is a critical component of the Northeast China Tiger and Leopard National Park, which includes the Jilin Hunchun Amur Tiger National Nature Reserve. Chunhua Town spans 2082 km^2^ and experiences a mesothermal near-oceanic monsoon climate, with a mean annual temperature of 7 °C and a mean annual precipitation of 651 mm. The area is rich in wildlife resources and is a highly suitable habitat for the Amur tiger, as well as the Amur leopard (*Panthera pardus orientalis*) and sika deer (*Cervus nippon*). Wild boars are abundant in the area, posing a significant threat to maize (*Zea mays*). The most severe damage occurs during the critical 30-day maturation period spanning from the milky stage to harvest-ready stage in September.

### 2.2. Field Testing of Countermeasures

Experimental plots were established in flat cornfields adjacent to broadleaf forests, which had experienced significant wild boar damage (Figure 1). Each experimental farmland plot covered the effective prevention area of the devices—a 200 m radius semicircle facing the forest, with its forest-proximal edge within 100 m of the forest boundary. The vegetation along the forest edge was predominantly composed of broadleaf trees, including birch and linden. On the non-forest side, the plots were primarily bordered by rivers and roads, though some were also adjacent to cornfields. As most wild boars entered the cornfields from the forest edge, all equipment, except for the electric fence and anti-animal ribbons, was placed on the side closest to the forest, oriented toward the forest edge. The electric fence was installed to enclose the entire plot perimeter, while the anti-animal ribbons were installed to encircle the outer boundary of the farmland.

To control for confounding variables, including soil type, elevation gradient, climate, and distance from forest edges, all experimental plots (2016–2021) were established within homogeneous areas sharing comparable edaphic climate conditions and topographical conditions. The deployment of all countermeasures consistently adhered to the criteria established in the first year across subsequent years and we assumed that changes in wild boar population dynamics had no direct impacts, as the study site was chosen from areas with consistent wild boar-related damage. Three sample plots and three control plots were set up for each treatment. The control plots were established near the experimental plots, with no treatment or intervention applied. To avoid spatial interference among countermeasures, the distance between different countermeasures was no less than 200 m.

Different treatments were tested in different years (Table 1), with most treatments tested over three to four years. This multiple testing method allowed us to analyze whether treatments were equally effective when applied a second time (temporal efficacy).

### 2.3. Wild Boar Damage Monitoring

Wild boar damage mainly occurs from the evening to the early morning of the next day. Therefore, monitoring for wild boar damage occurred at 7:00 a.m. every day in the cornfield where the countermeasure had been placed previously and in the experimental control plots. Data collection followed a standardized protocol, with two observers recording the presence or absence of damage to ensure consistency and reliability. Damage was recorded as a binary measure for each plot, where damage was considered present or absent if fresh boar tracks, nibbling, or trampling of corn occurred in any area within 50 m from where the countermeasure was placed. The observation period spanned from the ‘milk’ stage of corn development to harvest maturity, lasting 30 to 40 days.

### 2.4. Deployment of Wild Boar Countermeasures

Wild boar countermeasures were categorized into five groups: visual, auditory, tactile, and olfactory groups, based on the sensory stimuli employed, as well as a composite group.

- Visual Group

**Figure A1:**
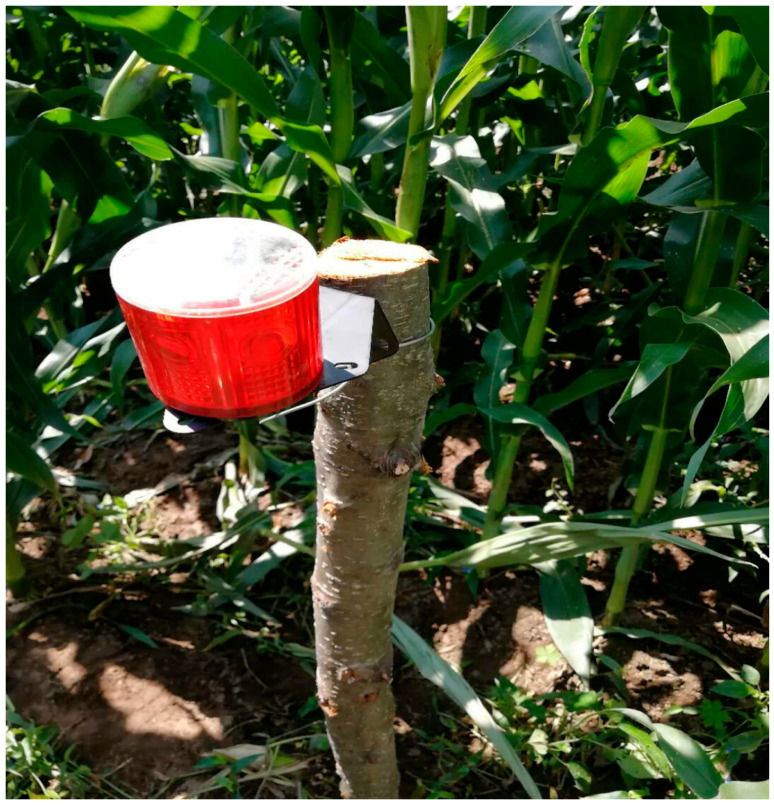
Setting mode of solar blinker in farmland.

Solar blinkers and anti-animal ribbons were used in this experimental group. We placed solar blinkers with varied colors at a height of 1.7 to 2.0 m on posts (Table 1). As well, anti-animal ribbons were also attached to posts and placed surrounding the outer perimeter of the farmland. These were made of plastic film and fibers, showing aluminum foil or a fuchsia color, and moved in the wind. The solar blinkers and anti-animal ribbons were positioned at least 100 m apart, with each set placed at the center of the experimental plots (Figure A1).

- Auditory Group

**Figure A2:**
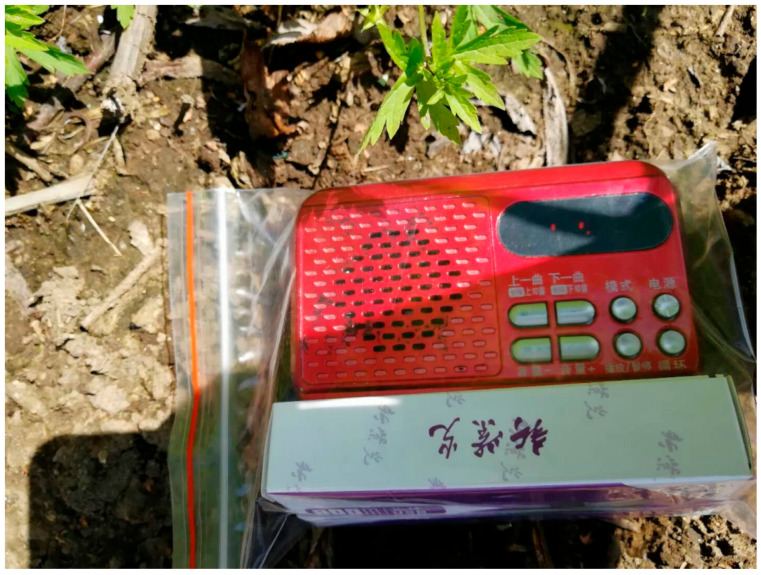
Recorder with rainproof shade.

Sounds of Amur tiger calls (natural sound), wild boar distress calls, and wolf calls (natural sound) were used in this group. These sounds were recorded from the Harbin Northeast Tiger Forest Park, downloaded from the public website “http://sc.chinaz.com/ (accessed on 13 June 2016)”, and adjusted to specific durations to fit different time intervals (Table 1). Supplementary Amur tiger vocalizations, including adult and juvenile calls, were field recorded at Harbin Northeast Tiger Forest Park using smartphones. In the experimental plots, they were played during dusk, dawn, and nighttime using high-power loudspeakers (Figure A2).

- Tactile Group

**Figure A3:**
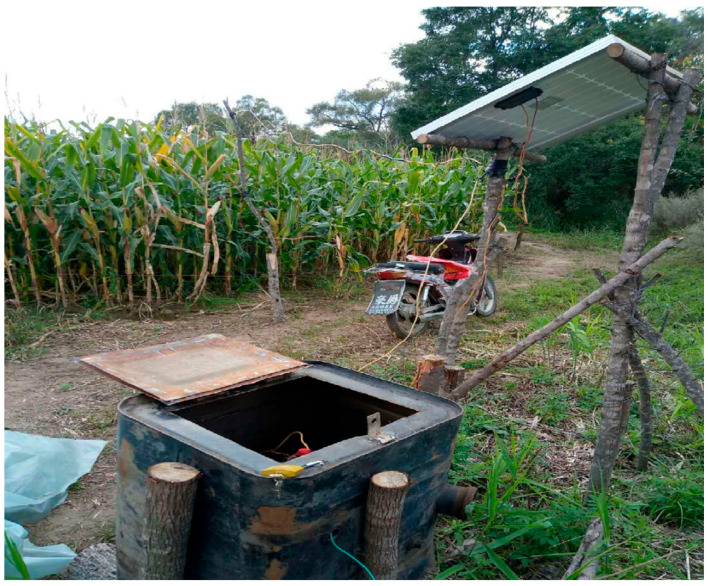
Electric fencing.

Pulsed electric fences were installed around available cornfields. We tested and compared fences with varied coils which altered the strength of current (Table 1, Figure A3).

- Olfactory Group

**Figure A4:**
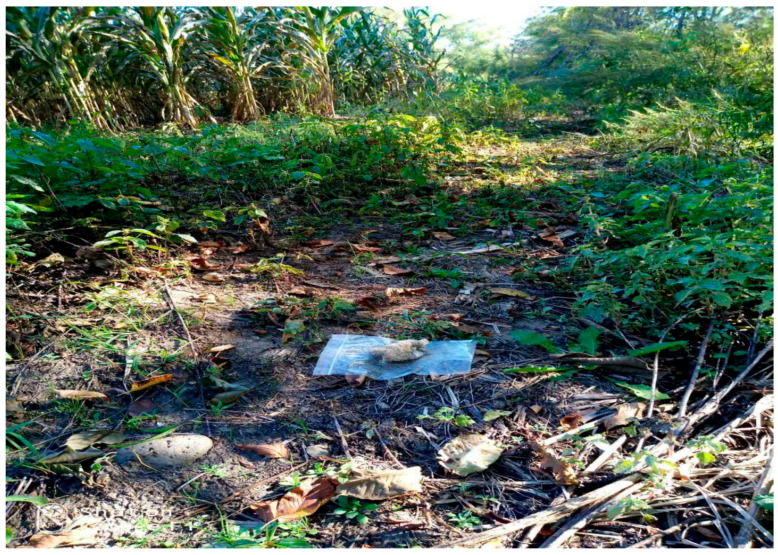
Adult Amur Tiger feces with rainproof shade.

Fresh Adult Amur tiger feces were collected from the Harbin Tiger Forest Park, carried in a plastic bag and encapsulated in a two-layer Ziplock bag. About 0.5 kg of feces were scattered along a 20 m section at the edge of the field on the side where wild boars entered the farmland and replaced every 3 days (Table 1, Figure A4).

- Combined Group

This group employed a combination of deploying Adult Amur tiger feces and calls (Table 1). The application of these countermeasures followed both methods outlined for the auditory and olfactory groups.

### 2.5. Temporal Efficacy Analysis

The temporal continuity of countermeasures was assessed by examining the duration of their effectiveness over time. The validity period of wild boar deterrents was defined as the time from the start of the experiment until the first entry of wild boars into the plot. Trends in effectiveness were identified by analyzing year-on-year variations in validity periods, with patterns of increases, declines, stability, or fluctuations categorized accordingly. A reliable temporal analysis was not feasible for countermeasures with insufficient data (i.e., fewer than three years of data or data from specific conditions).

### 2.6. Data Analysis

The mean validity periods of the same countermeasures implemented across different sample plots within the same year were calculated to analyze the temporal continuity of each countermeasure’s effect.

Due to the small sample size and non-normal data distribution, the analysis was first conducted at the group level using the Kruskal–Wallis *H* test to assess differences among visual, auditory, tactile, olfactory, and combined treatments. Subsequently, the Mann–Whitney *U* test was applied to compare individual countermeasures with each other to identify the most effective one, as well as to compare all countermeasures against the control group (Table 1).

To calculate the cost-effectiveness of the most effective countermeasures, the repellency per cost ratio was defined to determine which countermeasure provides the best wild boar repellency relative to its installed cost. The mean validity period for each year was calculated by averaging the effectiveness duration across all experimental plots. These yearly mean values were then summed over the total study period to obtain the cumulative validity period. The total cost accounted for all expenditures incurred over the entire duration of the experiment. In the following formula,

$$Repellency\;per\;cost=\frac{Validity\;Period\;(mean)}{\mathrm{Total}\;Cost\;(IUS\$)}$$

The validity period is presented as mean ± standard deviation. For non-parametric statistical tests, results are reported using mean rank, and variance is expressed as the interquartile range.

All statistical analyses were performed using SPSS 26.0, while ArcGIS was used to create the sampling plot arrangement map. Bar graphs were generated in Origin 2024b.

## 3. Results

### 3.1. Effectiveness of Wild Boar Deterrence

The validity period of the control plots, where no countermeasures were applied, showed slight yearly fluctuations. In 2016 and 2017, the mean validity period was (2.43 ± 0.58) days. This decreased to (1.33 ± 0.42) days in 2018 and (1.67 ± 0.37) days in 2019, followed by a slight recovery to (2.14 ± 0.58) days in 2020 and (2.24 ± 0.58) days in 2021.

**Figure 2:**
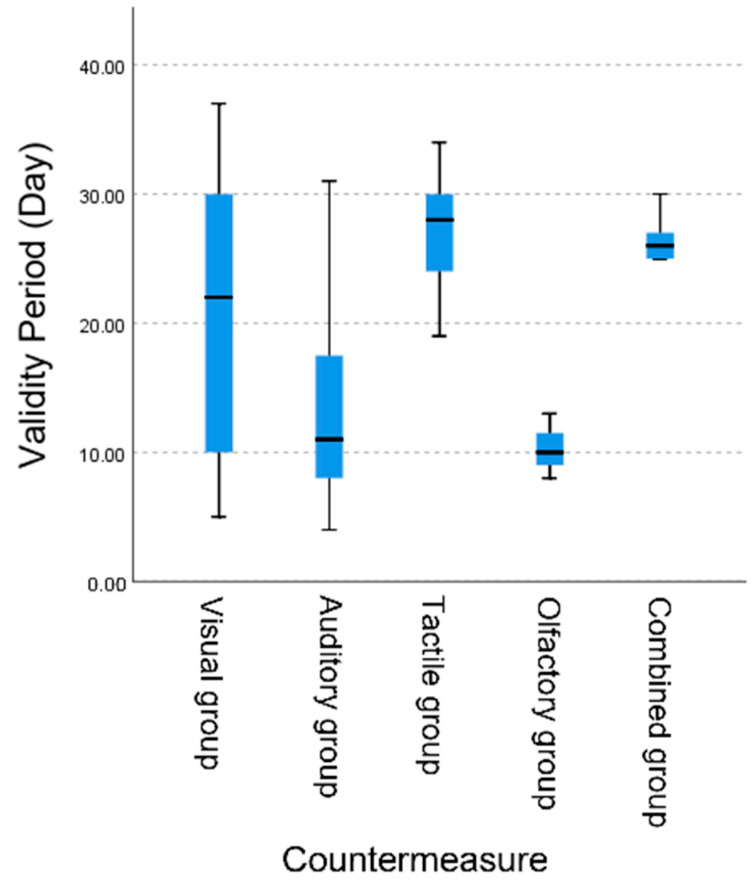
Countermeasure effectiveness by sensory group: Kruskal–Wallis *H* Test for visual, auditory, tactile, olfactory, and combined measures. Error bars represent interquartile range (IQR). Mean ranks for each group: visual (118.29), auditory (72.60), tactile (152.56), olfactory (59.83), and combined (145.17). Sample numbers: visual (*n* = 69), auditory (*n* = 96), tactile (*n* = 27), olfactory (*n* = 3), and combined measures (*n* = 6).

The results of the Kruskal–Wallis *H* test showed that the tactile group had the highest mean rank of 152.56, followed closely by the visual group with a mean rank of 118.29. In contrast, the olfactory group had the lowest mean rank, at only 18.67 (Figure 2).

All countermeasures showed significant differences compared to the control group (*p* < 0.05) (Table A1).

**Figure 3:**
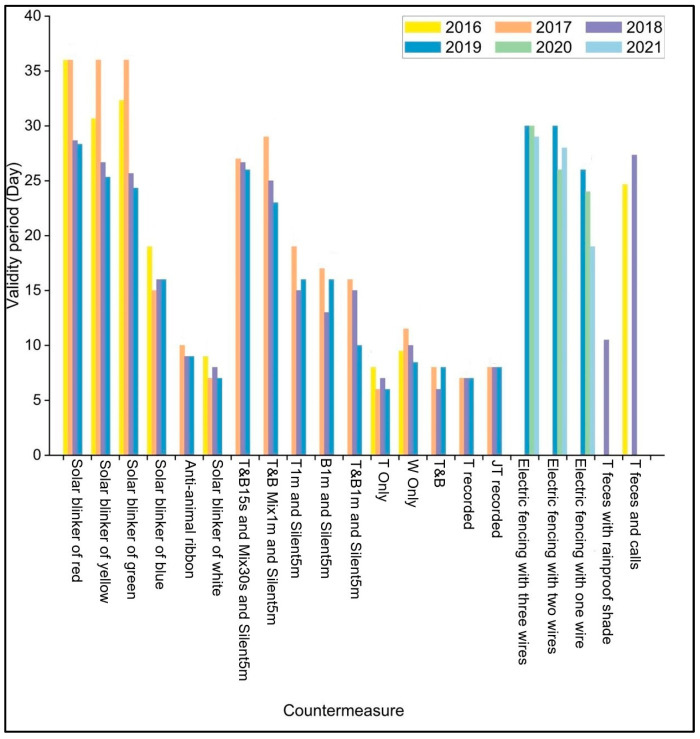
Validity period of wild boar damage. Validity period of countermeasures’ effectiveness varies over time represents the mean of three experimental plots. Lowercase letters indicate significant differences between each countermeasure. Each countermeasure was tested across three experimental plots. Name of countermeasure refers to Table 1. Legend represents year in which the countermeasure was implemented.

The validity periods of the 1000 mA red, yellow, and green solar blinkers (treatments A, B, and C) were significantly greater than those of the 1000 mA white and blue solar blinkers and the anti-animal ribbons (*p* < 0.05), with no significant differences observed among the red, yellow, and green solar blinkers themselves (*p* > 0.05) (Figure 3, Table A2).

For the auditory group, the T&B15s and Mix30s and Silent5m, and T&B Mix1m and Silent5m both significantly outperformed the other auditory countermeasures (*p* < 0.05). However, no significant difference was observed between the two best-performing auditory countermeasures (*p* > 0.05), making them the most effective auditory countermeasures (Figure 3, Table A3).

For the tactile group, the electric fencing with two and three wires significantly outperformed the electric fencing with one wire in the validity period (*p* < 0.05), with no significant difference between the two and three wire countermeasures (*p* > 0.05). Thus, the electric fencing with two and three wires were the most effective countermeasures for this group (Figure 3, Table A4).

In the olfactory and combined categories, the use of adult Amur tiger feces with a rainproof shade and adult Amur tiger feces and calls emerged as the most effective countermeasures in each group.

Across all groups, the best-performing countermeasures were the 1000 mA red, yellow, and green solar blinkers; T&B15s and Mix30s and Silent5m; electric fencing with two and three wires; and adult Amur tiger feces and calls (Table A5). The validity periods of the T&B Mix1m and Silent5m, as well as the adult Amur tiger feces with a rainproof shade, were significantly lower than those of the other best countermeasures (*p* < 0.05).

### 3.2. Temporal Dynamics of Countermeasure Efficacy

In the analysis of the temporal continuity of the countermeasures, specific countermeasures, like the 1000 mA red, yellow, and green solar blinkers, T&B Mix1m and Silent5m, and T&B1m and Silent5m, exhibited a significant downward trend in their effectiveness as the years progressed. In contrast, some strategies, such as the anti-animal ribbon, T&B15s and Mix30s and Silent5m, T and JT recorded, and electric fencing with three wires, maintained a consistent effectiveness duration (Figure 3). Other countermeasures displayed fluctuating trends, with no clear annual trend. The limited data on the adult Amur tiger feces with a rainproof shade and adult Amur tiger feces and calls (only one year) prevented the assessment of their temporal continuity.

### 3.3. Cost-Effectiveness Analysis of Wild Boar Countermeasures

**Figure 4:**
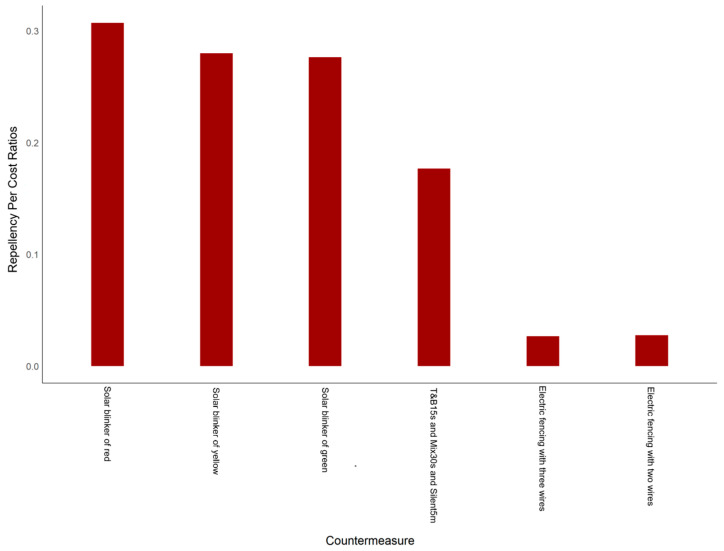
Comparative analysis of repellency per cost ratios for most effective countermeasures. Name of countermeasure refers to Table 1.

The results of the cost-effectiveness analysis showed that the 1000 mA red solar blinkers, with a value of 30.29 IUS$/hm^2^, had the highest repellency per cost ratio of 0.31, making them a highly economical choice with a relatively long validity period. The repellency per cost ratios for the 1000 mA yellow and green solar blinkers were slightly lower, and both were 0.28. Conversely, the electric fencing with three wires, with a value of 319.69 IUS$/hm^2^, demonstrated the lowest repellency per cost ratio at 0.027, while the electric fencing with two wires had a slightly higher ratio of 0.028, despite its more extended validity period (Figure 4).

## 4. Discussion

Given the minimal year-to-year variation in the validity period of the control group, the influence of extraneous factors beyond the countermeasures can be considered negligible. Among the tactile deterrents, electric shocks exhibit the most substantial and prolonged effect on wild boar behavior compared to other deterrent methods. As a painful stimulus, an electric shock effectively induces and reinforces avoidance behavior in wildlife [34]. For example, Linhart et al. found that three to five electric shocks conditioned captive coyotes (*Canis latrans*) to avoid black rabbits for three to nine months [35]. Similarly, red foxes (*Vulpes vulpes*) continued avoiding areas once enclosed by electric fencing even after its removal [36], and goats (*Capra hircus*) avoided electrified fences and the fence posts where they once stood [37]. These findings provide strong evidence for the long-term efficacy of electric shocks in sustaining avoidance behavior in wildlife [38].

Among the tactile group, electric fencing with three wires proved to be highly effective and durable option for preventing wild boar damage, with the protection levels scaling positively with wire number. Wild boars developed avoidance behaviors after associating fence contact with pain. Schlageter and Daniel demonstrated that pain, rather than fear, primarily drives this avoidance [27]. Over time, wild boars formed a conditioned response linking electric shocks to the fence [33].

The visual group showed moderately lower effectiveness than the tactile group, potentially attributable to the inclusion of 1000 mA blue and white solar blinkers, as well as the suspension of anti-animal ribbons, with both showing short validity periods. Specifically, the 1000 mA red, yellow, and green solar blinkers effectively deterred wild boars, as their emitted wavelengths (465–580 nm) align with boar visual sensitivity [38]. These solar blinkers initially suppressed nocturnal boar activity, similar to Linhart’s findings, where solar blinkers decreased coyote predation on sheep by 95% over 53 days [39]. However, habituation diminished their effectiveness after two years [29].

Additionally, Schlageter et al. reported only 8.1% effectiveness for solar blinkers in preventing farmland entry by wild boars, concluding their insufficiency for reliable crop protection [23]. In their experimental design, food bait consumption near blinkers was monitored to assess deterrence. However, the one-year study duration and methodological differences may explain these discrepancies in the results.

In addition, T&B15s and Mix30s and Silent5m or adult Amur tiger feces and calls also proved to be effective deterrents, likely due to the wild boars’ innate predator avoidance instincts [40]. The resurgence of the Amur tiger population is closely tied to the effectiveness of wild boar management [41]. Under conservation initiatives like the “Tianbao Project” [42], the wild Amur tiger population surpassed 55 individuals by 2016 [43] and continues to grow [44], potentially amplifying the long-term utility of tiger-derived stimuli.

The Cost-effective analysis revealed that 1000 mA red solar blinkers are the most economical short-term solution, with a repellency per cost ratio of 0.31 at only 30.29 IUS$/hm^2^—though their effectiveness declines after two years. Electric fencing with three wires, while costly and with a lower cost-effective ratio of 0.027, provides decade-long protection with proper maintenance [45], justifying its use for sustained defense. Therefore, 1000 mA red solar blinkers are suitable for short-term use, while electric fencing with three wires offers superior durability and long-term protection.

Human–wild boar conflicts, primarily resulting from crop destruction [1], can be mitigated with various countermeasures, such as solar blinkers, adult Amur tiger calls, and feces, effectively reducing damage. However, habituation may reduce their efficacy over time. Integrating electric fencing with technologies like video surveillance, perimeter alarms, electronic patrol systems, and SMS alert systems enhances monitoring and responses to improve long-term deterrence [46], This setup allows the real-time transmission of data, including wild boar movements and alarm signals, to relevant management personnel, ensuring timely interventions.

## 5. Conclusions

This study underscores the variable effectiveness of sensory deterrents in influencing wild boar behavior. Tactile deterrents, particularly electric shocks, were the most effective in eliciting long-term avoidance, indicating the wild boars’ sensitivity to painful stimuli. While the short-term cost-effectiveness of these deterrents is relatively low, their extended durability, lasting as long as ten years, renders them a viable long-term management strategy. In contrast, visual deterrents, such as solar blinkers, provide an economically efficient solution in the short term but exhibit diminished effectiveness over time due to habituation. Auditory cues, such as predator calls, also provoke avoidance, emphasizing the importance of perceived threats.

These findings highlight the potential of sensory-based deterrents; however, their practical application requires a nuanced integration of timing, ecological context, and technical rigor to achieve sustainable outcomes. To bridge the gap between empirical evidence and field implementation, the following management recommendations are proposed based on the interplay of behavioral responses and environmental variables observed in this study.

- Timely deployment: Wild boar activity peaks during corn’s grain-filling and maturation stages (September), necessitating preventive measures (e.g., electric fences) be installed at least two weeks in advance to establish conditioned avoidance.
- Technical standardization: Farmer-led installations often lack compliance, advocating for centralized procurement and maintenance by local authorities to ensure efficacy.
- Context-specific deterrence: In regions devoid of Amur tigers, reliance on tiger-derived cues (feces, calls) is ineffective. However, in tiger-present areas, combining these cues with supplementary deterrents (e.g., wolf calls) may amplify effectiveness by leveraging innate predator avoidance behaviors.
- Agricultural diversification: Planting less palatable crops (e.g., chili peppers or ginger) or intercropping could reduce susceptibility.
- Habitat restoration: Strategic farmland-to-forest conversion in high-conflict zones may buffer human–wildlife interactions.
- Economic alternatives: Hybrid wild boar breeding programs and ecotourism initiatives could alleviate reliance on natural populations while fostering coexistence.

Future research should focus on investigating the synergistic effects of multi-modal deterrence, examining the impacts of habituation, and refining cost-effectiveness strategies to support the development of sustainable, long-term wildlife management approaches.

## Figures and Tables

**Table 1:** Experimental treatments are used to investigate the validity of period deterrents to wild boar damage in cornfields of Chunhua Town. Cost of deployment and years in which these countermeasures were tested are also indicated. Cost values in international dollars (IUS$) were converted from Chinese Yuan (CNY) using the 2025 purchasing power parity (PPP) exchange rate of USD 1 = CNY 3.466, based on data from CEIC. For the auditory group, abbreviations in parentheses indicate names of each countermeasure: T = Adult Amur tiger, JT = Juvenile Amur tiger, B = Wild boar, and W = Wolf.

Group		Countermeasure	Total Sample Numbers (*n*)	Cost (IUS$)	Year
Visual group	A	1000 mA red solar blinker	12	30.29/hm^2^	2016~2019
	B	1000 mA yellow solar blinker	12	30.29/hm^2^	2016~2019
	C	1000 mA green solar blinker	12	30.29/hm^2^	2016~2019
	D	1000 mA blue solar blinker	12	30.29/hm^2^	2016~2019
	E	Suspension of anti-animal ribbon	9	5.19/piece	2017~2019
	F	1000 mA white solar blinker	12	43.28/hm^2^	2016~2019
Auditory group	G	Adult Amur tiger calls 15 s and wild boar calls 15 s plus combined 30 s plus blank for 5 min (T&B15s and Mix30s and Silent5m)	9	43.28/hm^2^	2017~2019
	H	Combined adult Amur tiger calls and wild boar calls for 1 min plus blank for 5 min (T&B Mix1m and Silent5m)	9	43.28/hm^2^	2017~2019
	I	Adult Amur tiger calls for 1 min plus blank for 5 min (T1m and Silent5m)	9	43.28/hm^2^	2017~2019
	J	Wild boar calls for 1 min plus blank for 5 min (B1m and Silent5m)	9	43.28/hm^2^	2017~2019
	K	Adult Amur tiger calls and wild boar calls for 1 min plus blank for 5 min (T&B1m and Silent5m)	9	43.28/hm^2^	2017~2019
	L	Adult Amur tiger calls (T Only)	12	43.28/hm^2^	2016~2019
	M	Wolf calls (W Only)	12	43.28/hm^2^	2016~2019
	N	Adult Amur tiger calls and wild boar calls (T&B)	9	43.28/hm^2^	2017~2019
	O	Adult Amur tiger calls recorded (T recorded)	9	43.28/hm^2^	2017~2019
	P	Juvenile Amur tiger calls recorded (JT recorded)	9	43.28/hm^2^	2017~2019
Tactile group	Q	Electric fencing with three wires	9	319.69/hm^2^	2019~2021
	R	Electric fencing with two wires	9	292.05/hm^2^	2019~2021
	S	Electric fencing with one wire	9	264.27/hm^2^	2019~2021
Olfactory group	T	Adult Amur tiger feces with rainproof shade	3	5.77/piece	2018
Combined group	U	Adult Amur tiger feces and calls	3	49.04/hm^2^	2018

## Data Availability

All data used in this research have been obtained with the explicit consent of the original researchers. Our study builds upon previous research and utilizes their raw data for further analysis. Furthermore, our research and the original study are part of the same funding project, ensuring alignment in the objectives and ethical compliance.

## Appendix A

**Table A1:** Comparison of all countermeasures with control group.

**Number**		**A**	**B**	**C**	**D**	**E**	**F**	**G**	**H**	**I**	**J**	**K**
Control	*p*	0.000	0.000	0.000	0.000	0.000	0.000	0.000	0.000	0.000	0.000	0.000
	Z	−4.220	−4.220	−4.218	−4.220	−3.625	−4.211	−3.633	−3.627	−3.623	−3.623	−3.625
**Number**		**L**	**M**	**N**	**O**	**P**	**Q**	**R**	**S**	**T**	**U**	
Control	*p*	0.000	0.000	0.000	0.000	0.000	0.000	0.000	0.000	0.046	0.004	
	Z	−4.222	−4.227	−3.631	−3.631	−3.643	−3.629	−3.686	−3.684	−1.993	−2.918	

**Table A2:** Comparison of repellent effects of visual group of countermeasures.

Number		A	B	C	D	E
B	*p*	0.137				
	Z	−1.487				
C	*p*	0.162	0.930			
	Z	−1.399	−0.087			
D	*p*	0.001	0.001	0.001		
	Z	−3.393	−3.393	−3.389		
E	*p*	0.009	0.009	0.009	0.018	
	Z	−2.614	−2.614	−2.610	−2.374	
F	*p*	0.001	0.001	0.001	0.003	0.421
	Z	−3.389	−3.389	−3.386	−2.923	−0.805

**Table A3:** Comparison of repellent effects of auditory group of countermeasures.

Number		G	H	I	J	K	L	M	N	O
H	*p*	0.064								
	Z	−1.850								
I	*p*	0.002	0.004							
	Z	−3.122	−2.846							
J	*p*	0.003	0.003	0.079						
	Z	−2.948	−2.928	−1.759						
K	*p*	0.013	0.014	0.085	0.651					
	Z	−2.478	−2.449	−1.722	−0.453					
L	*p*	0.000	0.000	0.087	0.559	0.716				
	Z	−3.720	−3.558	−1.710	−0.584	−0.364				
M	*p*	0.001	0.001	0.013	0.107	0.288	0.391			
	Z	−3.479	−3.468	−2.484	−1.611	−1.206	−0.857			
N	*p*	0.002	0.002	0.004	0.006	0.018	0.538	0.856		
	Z	−3.133	−3.116	−2.918	−2.770	−2.374	−0.615	−0.181		
O	*p*	0.002	0.002	0.004	0.006	0.019	0.040	0.171	0.039	
	Z	−3.122	−3.105	−2.903	−2.751	−2.343	−2.049	−1.369	−2.060	
P	*p*	0.013	0.014	0.019	0.024	0.046	0.009	0.012	0.017	0.019
	Z	−2.484	−2.455	−2.353	−2.249	−1.993	−2.617	−2.509	−2.384	−2.353

**Table A4:** Comparison of repellent effects of tactile group of countermeasures.

Number		Q	R
R	*p*	0.246	
	Z	−1.159	
S	*p*	0.046	0.047
	Z	−1.993	−1.771

**Table A5:** Comparison of repellent effects among best countermeasures.

Number		A	B	G	H	Q	R	T
B	*p*	0.137						
	Z	−1.487						
C	*p*	0.162	0.930					
	Z	−1.399	−0.087					
G	*p*	0.002	0.082					
	Z	−3.154	−1.740					
H	*p*	0.000	0.001	0.003				
	Z	−4.070	−3.453	−3.020				
Q	*p*	0.380	0.663	0.335	0.001			
	Z	−0.877	−0.436	−0.743	−2.586			
R	*p*	0.126	0.716	0.307	0.027	0.246		
	Z	−1.531	−0.364	−1.020	−2.206	−1.159		
T	*p*	0.001	0.001	0.001	0.001	0.002	0.002	
	Z	−3.384	−3.384	−3.384	−3.464	−2.334	−2.324	
U	*p*	0.038	0.322	0.505	0.001	0.291	0.791	0.004
	Z	−2.080	−0.991	−0.666	−2.560	−1.055	−0.265	−2.887
